# Long-Term Outcomes of External Dacryocystorhinostomy in the Age of Transcanalicular Microendoscopic Techniques

**DOI:** 10.1155/2016/5918457

**Published:** 2016-03-24

**Authors:** M. Alnawaiseh, N. Mihailovic, A. C. Wieneke, V. Prokosch, A. Rosentreter, R. L. Merté, N. Eter

**Affiliations:** Department of Ophthalmology, University of Muenster Medical Center, 48149 Muenster, Germany

## Abstract

*Purpose.* This study aimed to evaluate long-term results of external dacryocystorhinostomy (DCR) at a tertiary eye care center specializing in lacrimal duct surgery in Germany.* Methods.* The medical records of 1010 patients with acquired nasolacrimal duct obstruction (NLDO), who had undergone lacrimal duct surgery at a tertiary eye care center, were reviewed. Only adult patients who had undergone external DCR were included. The evaluation included the following parameters: age, gender, duration of symptoms, patient satisfaction, previous dacryocystitis, complication rates, and surgical outcome.* Results.* 154 eyes of 146 patients (14.5%) could be included in the study. The average age was 64.1 ± 29.7 years. 66.4% of patients were females and 33.6% were males. Acute or chronic dacryocystitis was found in 81 patients (55.5%). Overall, 82.8% of patients had full resolution of symptoms. The success rate of external DCR for patients with previous episodes of dacryocystitis was 82.7% compared to 83.4% for patients without dacryocystitis in their medical history.* Conclusion.* In cases in which transcanalicular microendoscopic techniques are contraindicated (e.g., after dacryocystitis) or in complex cases where microendoscopic procedures have failed (revision surgery), external DCR is still the surgical treatment of choice with very good postoperative success.

## 1. Introduction

Acquired nasolacrimal duct obstruction (NLDO) is a common disorder that occurs more frequently in females [1, 2]. Epiphora is the most common symptom.

Dacryocystorhinostomy (DCR) is the standard treatment for nasolacrimal duct obstruction. There are two main types of DCR: external and endonasal. External DCR was first described by Toti in 1904 [3], and the procedure has been modified many times by different surgeons over the years [1]. The endonasal technique was first described by West in 1910 [4].

However, since the early 1990s minimally invasive microendoscopic transcanalicular therapeutic techniques such as laser dacryoplasty (LDP) or microdrill dacryoplasty (MDP) have become more and more popular [5, 6]. These procedures allow the physiology of the lacrimal drainage system to be preserved intact and obviate the need for an external DCR. Thus, in specialized centers the number of external DCRs performed has decreased markedly, and external DCR is usually chosen only when transcanalicular microendoscopic techniques are contraindicated, such as in revision operations or in complicated or traumatic cases [5–8].

The purpose of the current study was to evaluate preoperative characteristics and postoperative outcomes of external DCR in this new and demanding indication area at a tertiary referral center specializing in lacrimal duct surgery in Germany.

## 2. Materials/Subjects and Methods

In this study we retrospectively reviewed the medical records of all patients who had undergone lacrimal surgery at a tertiary eye care center between 2009 and 2011. The retrospective study was approved by the local ethics committee and adhered to the tenets of the Declaration of Helsinki.

### 2.1. Inclusion Criteria

Inclusion criteria are as follows:

1. adult patients (age over 18 years);
2. patients with acquired NLDO;
3. patients s/p external DCR performed at our department between 2009 and 2011.

The extracted data included patient demographics, side of obstruction, duration of symptoms, previous dacryocystitis, previous lacrimal duct surgery, postoperative complications, and surgical outcome.

### 2.2. Surgical Procedure

In our department external DCR is generally performed under general anesthesia. An incision was made medial to the angular vein at the level of the medial canthal ligament. An osteotomy with a mean diameter of 10 mm was created and the lacrimal sac opened. Posterior and anterior mucosal flaps were made and all patients were intubated with silicone tubes. The skin was closed with a 6-0 polypropylene suture. The silicon tubes were usually kept in place for 3–6 months.

Postoperative, long-term results regarding patient satisfaction and success rate were evaluated by telephone survey in October 2015. Success was defined as full resolution of symptoms and no postoperative dacryocystitis without additional postoperative lacrimal duct surgery. Regarding the analysis of long-term outcome, only the first side was included in bilateral cases. Patients were also asked to rate their satisfaction on a scale of 1 to 10 (1 = extremely dissatisfied to 10 = extremely satisfied).

Data management was performed with Microsoft Excel 2010. IBM SPSS® Statistics 22 for Windows (IBM Corporation, Somers, NY, USA) was used for statistical analyses. Fisher's exact test was used to compare the results of DCR surgeries in the different subgroups.

## 3. Results

**Figure 1:**
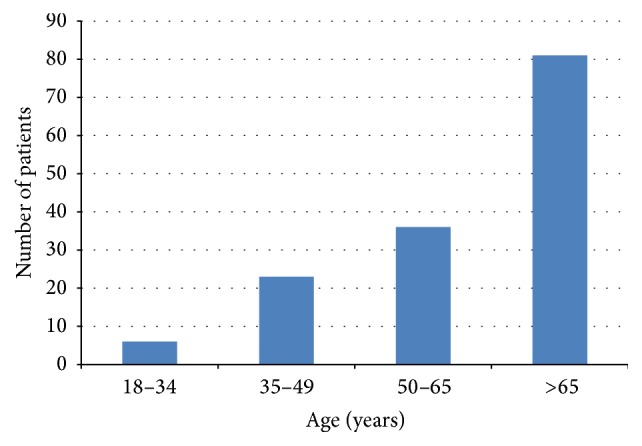
Patient age.

From the medical records of a total of 1010 patients who had undergone lacrimal duct surgery (*n* = 1361) from January 2009 to December 2011, 154 eyes of 146 patients (14.5%) were included according to the previously mentioned inclusion criteria. Mean preoperative age was 64.1 ± 29.7 years; 97 patients (66.4%) were females and 49 patients (33.6%) males. Demographics of the study population are summarized in Table 1. 81 patients (55.5%) were older than 65 years (Figure 1). 138 had unilateral DCR (right side, 75 (51.4%); left side, 63 (43.2%)), and 8 patients had bilateral DCR.

**Figure 2:**
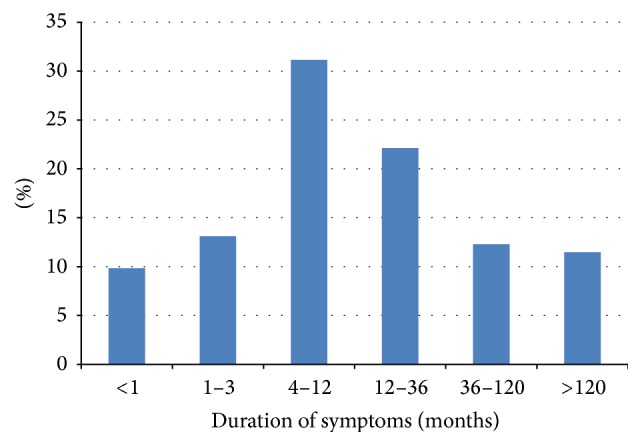
Duration of symptoms.

Figure 2 shows the duration of symptoms. The majority of the patients showed a duration of symptoms prior to first presentation at our department of more than one year (Figure 2). 78.8% (*n* = 115) patients complained of epiphora, and 55.5% (*n* = 81) of patients had previously had acute or chronic dacryocystitis.

97 of the 146 patients (66.4%) underwent DCR as the initial lacrimal duct surgery. 33.6% of patients (*n* = 49) had had previous lacrimal duct surgery at another institution.

Telephone interviews could be performed in 87 patients (59.6%). The follow-up period ranged from 42 months to 80 months (mean 61.7 ± 5.1 months). The success rate of DCR in different subgroups is summarized in Table 2. The success rate of external DCR for patients with previous episodes of dacryocystitis was 82.7%, compared to 83.4% for patients without previous episodes of dacryocystitis. The difference was not statistically significant (*p* = 1.0). The success rate in patients without previous lacrimal duct surgery was 88.5% compared to 74.3% for patients with previous lacrimal duct surgery. The difference was also not statistically significant (*p* = 0.15).

**Figure 3:**
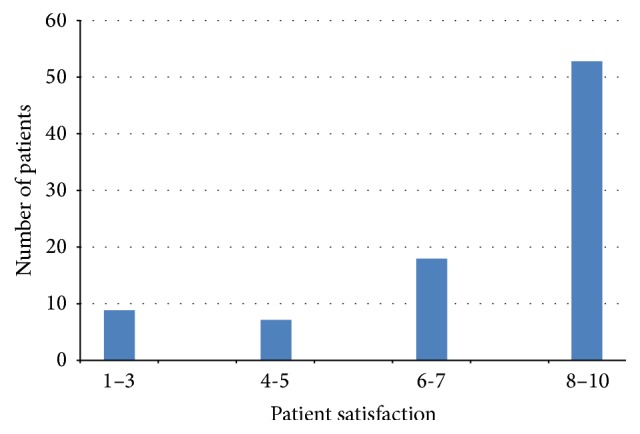
Patient satisfaction (1–10: 1 = extremely dissatisfied to 10 = extremely satisfied).

Patient satisfaction with the surgical outcome is summarized in Figure 3. Significant postoperative hemorrhage was observed in 2 cases (1.4%).

## 4. Discussion

The relative number of external DCR procedures in relation to the total number of all forms of lacrimal duct surgery performed at our department (14.5%) agrees with results from other centers specializing in lacrimal duct surgery [5, 7]. Due to the development of microendoscopic transcanalicular techniques (MDP and LDP) over the last 10 years, the relative number of external DCR procedures performed has decreased markedly [5–7]. The minimally invasive, scarless endoscopic techniques have an acceptable success rate of up to 80% and are highly suitable for use as an initial treatment (first-step procedure) [5–7]. In recent years external DCR at our department has been performed mainly following dacryocystitis, as a secondary procedure after prior lacrimal duct surgery, or where transcanalicular microendoscopic techniques were contraindicated [5–8].

66.4% of the patients were females, which is comparable with reports in the literature [1, 2, 9]. The majority of patients were older than 65 years. As DCR has a better success rate than transcanalicular microendoscopic techniques [1, 6, 7, 9–12], we applied DCR more frequently in elderly multimorbid patients to minimize the risk of revision surgery and further general anesthesia.

About 50% of patients showed duration of symptoms of less than one year prior to first presentation at the clinic. Some patients with chronic symptoms and a history of different forms of lacrimal duct surgery reported their symptoms to have lasted for several years.

External DCR in the present study has a success rate of 82.8%. The success rate of external DCR in the literature has been reported to lie between 80% and 99% [13–17].

Comparing success rates of lacrimal duct surgery is a difficult task because different studies use different success criteria (anatomic patency, improvement in tearing, or full resolution of symptoms) and the follow-up time varies widely. Evidence of anatomic patency to irrigation does not provide any information about the physiologic function of the DCR or patient satisfaction and can overestimate surgical success [18–20]. In this study only patients who became completely asymptomatic following DCR, with a minimum follow-up time of 3.5 years after surgery, were rated as successes. A further important aspect to take into consideration is the type of study population. This study investigated success rates of external DCR in some completely new and demanding indication areas: elderly patients (55.5% were older than 65 years), patients after dacryocystitis (55.5%), or patients after previous lacrimal duct surgery (33.6%).

In this study 55.5% of the patients had had a previous episode of dacryocystitis; there was no significant difference in the success rate of external DCR in patients with or without a previous episode of dacryocystitis. These findings are consistent with other studies in the literature [21].

Our study is limited by its retrospective design. However, the relatively large sample size with a long follow-up (mean follow-up of 61.7 ± 5.1 months) and the unusual study population (reflecting real-life data from a tertiary referral center specializing in lacrimal duct surgery) do add value to this study.

## 5. Conclusion

In conclusion, with the rapid development and progress achieved in minimally invasive microendoscopic lacrimal duct surgery, the relative number of external DCRs performed as first-line treatment has decreased markedly and the indication area has changed completely over the last 10 years. However, external DCR is still the surgical treatment of choice with very good postoperative success rate in special indication fields.

## Figures and Tables

**Table 1:** Baseline characteristics of the study population.

Age (years) (mean ± SD)	64.1 ± 29.7
Gender [female : male]	97 (66.4%) : 49 (33.6%)
History of epiphora	78.8% (*n* = 115)
History of dacryocystitis	55.5% (*n* = 81)
Trauma	7.5% (*n* = 11)
Previous lacrimal duct surgery	33.6% (*n* = 49)
Follow-up (months) (mean ± SD)	61.7 ± 5.1

**Table 2:** Success rates in the entire group and different subgroups.

	Success rates
*Entire group*	82.8
Patients with previous episodes of dacryocystitis	82.7
Patients without previous episodes of dacryocystitis	83.4
DCR as primary procedure	88.5
DCR after any form of initial lacrimal surgery	74.3

## References

[1] Emmerich K. H., Busse H., Meyer-Rüsenberg H. W. (1994). Dacryocystorhinostomia externa. Technique, indications and results. *Ophthalmologe*.

[2] Woog J. J. (2007). The incidence of symptomatic acquired lacrimal outflow obstruction among residents of Olmsted County, Minnesota, 1976-2000 (an American Ophthalmological Society thesis). *Transactions of the American Ophthalmological Society*.

[3] Toti A. (1904). Nuovo metodo conservatore di cura radicale delle soppurazioni croniche del sacco lacrimale (dacriocistorinostomia). *Clínica Moderna (Firenze)*.

[4] West J. M. (1914). A window resection of the nasal duct in cases of stenosis. *Transactions of the American Ophthalmological Society*.

[5] Meyer-Rüsenberg H.-W., Emmerich K.-H. (2010). Modern lacrimal duct surgery from the ophthalmological perspective. *Deutsches Arzteblatt*.

[6] Emmerich K.-H., Ungerechts R., Meyer-Rüsenberg H.-W. (2009). Microendoscopic tear duct surgery. *Ophthalmologe*.

[7] Emmerich K.-H., Emmerich G. M., Steinkogler F.-J., Ungerechts R., Meyer-Rüsenberg G., Meyer-Rüsenberg H.-W. (2010). How did lacrimal endoscopy influence lacrimal surgery?. *Klinische Monatsblatter fur Augenheilkunde*.

[8] Alnawaiseh M., Böhm M. R., Rosentreter A. (2015). Traumatic lacrimal duct stenosis: demographics and success rate of surgical procedures for secondary treatment. *Klinische Monatsblätter für Augenheilkunde*.

[9] Badhu B. P., Dulal S., Kumar S., Thakur S. K. D., Sood A., Das H. (2005). Epidemiology of chronic dacryocystitis and success rate of external dacryocystorhinostomy in Nepal. *Orbit*.

[10] Lee D. W., Chai C. H., Loon S. C. (2010). Primary external dacryocystorhinostomy versus primary endonasal dacryocystorhinostomy: a review. *Clinical and Experimental Ophthalmology*.

[11] Stemplewitz B., Amin S., Emmerich K.-H., Gesser-Wendt C., Meyer-Rüsenberg H.-W. (2015). Minimally invasive lacrimal duct surgery with a multimodal concept for functional lacrimal stenosis. *Klinische Monatsblatter fur Augenheilkunde*.

[12] Derya K., Demirel S., Doganay S., Orman G., Cumurcu T., Gunduz A. (2013). Endoscopic transcanalicular diode laser dacryocystorhinostomy: is it an alternative method to conventional external dacryocystorhinostomy?. *Ophthalmic Plastic and Reconstructive Surgery*.

[13] Horix D., Struck H. G. (2004). Long term patency rate of the external dacryocystorhinostomy. A retrospective study in the years 1991–2000 at the University Eye Hospital in Halle. *Ophthalmologe*.

[14] Huang J., Malek J., Chin D. (2014). Systematic review and meta-analysis on outcomes for endoscopic versus external dacryocystorhinostomy. *Orbit*.

[15] Mäntynen J., Yoshitsugu M., Rautiainen M. (1997). Results of dacryocystorhinostomy in 96 patients. *Acta Oto-Laryngologica, Supplement*.

[16] Shun-Shin G. A., Thurairajan G. (1997). External dacryocystorhinostomy—an end of an era?. *British Journal of Ophthalmology*.

[17] Warren J. F., Seiff S. R., Kavanagh M. C. (2005). Long-term-results of external dacryocystorhinostomy. *Ophthalmic Surgery Lasers and Imaging*.

[18] Rosen N., Ashkenazi I., Rosner M. (1994). Patient dissatisfaction after functionally successful conjunctivodacryocystorhinostomy with Jones tube. *American Journal of Ophthalmology*.

[19] Tarbet K. J., Custer P. L. (1995). External dacryocystorhinostomy: surgical success, patient satisfaction, and economic cost. *Ophthalmology*.

[20] Amin M., Moseley I. F., Rose G. E. (2000). The value of intubation dacryocystography after dacryocystorhinostomy. *British Journal of Radiology*.

[21] Rabina G., Golan S., Neudorfer M., Leibovitch I. (2013). External dacryocystorhinostomy: characteristics and surgical outcomes in patients with and without previous dacryocystitis. *Journal of Ophthalmology*.

